# Post-traumatic Cavernous Carotid Pseudoaneurysm with Delayed Epistaxis

**DOI:** 10.7759/cureus.3002

**Published:** 2018-07-19

**Authors:** Girish Menon, Ajay Hegde, Rajesh Nair

**Affiliations:** 1 Neurosurgery, Kasturba Medical College, Manipal, IND

**Keywords:** cavernous carotid aneurysm, cerebrovascular bypass, epistaxis, ec-ic bypass

## Abstract

Cavernous carotid aneurysms (CCAs) pose considerable dilemmas in management. Delayed post-traumatic epistaxis is a rare presentation of CCA. Clinically, the symptomatic triad of unilateral blindness, orbital fractures, and massive epistaxis is pathognomonic for internal carotid artery (ICA) pseudoaneurysm. The epistaxis is usually profound, intermittent, and life-threatening in nature. As most of these cases are initially seen by a physician, a high index of suspicion is essential during its early identification. Traumatic aneurysms are pseudoaneurysms with a fibrous wall that rupture and cause massive epistaxis resulting from disruption through the sphenoid sinus wall. We report a young adult who presented with the triad and severe anemia four months following head injury. He was treated with ligation of the carotid artery and a high-flow extracranial-intracranial (EC-IC) bypass. In the era of endovascular coiling and flow diverters, EC-IC bypass still has a role in the treatment of complex giant aneurysms with comparable results.

## Introduction

Traumatic pseudoaneurysms of the cavernous carotid artery can be a challenging clinical problem, both in diagnosis and management. This case report describes a young adolescent boy with delayed epistaxis following traumatic brain injury sustained four months prior to presentation. Investigations revealed a pseudoaneurysm of the left cavernous carotid artery, which was surgically managed. The pitfalls in diagnosis and the management dilemmas are discussed.

## Case presentation

A 19-year-old boy was admitted with a history of recurrent bouts of epistaxis from his right nostril, for a duration of one month. The last episode was severe and uncontrolled, which prompted him to seek medical attention. His past medical history involved a motor vehicle accident four months prior to admission. This event was associated with a brief period of loss of consciousness, vomiting, and associated nasal bleed. He had no history of seizures or any cerebrospinal fluid (CSF) leak, but suffered a complete loss of vision in his left eye. Computed tomography (CT) of the brain revealed a displaced fracture of the left frontal bone with small underlying extradural hematoma with fracture of the orbital roof and spheno ethmoid sinus. He was managed conservatively with antiepileptics, antibiotics and closely observed for CSF leak. Steroids were administered for his left traumatic optic neuropathy and he was discharged after two weeks of observation. On discharge, his Glasgow coma scale was 15 and he had no perceivable vision in his left eye.  

**Figure 1:**
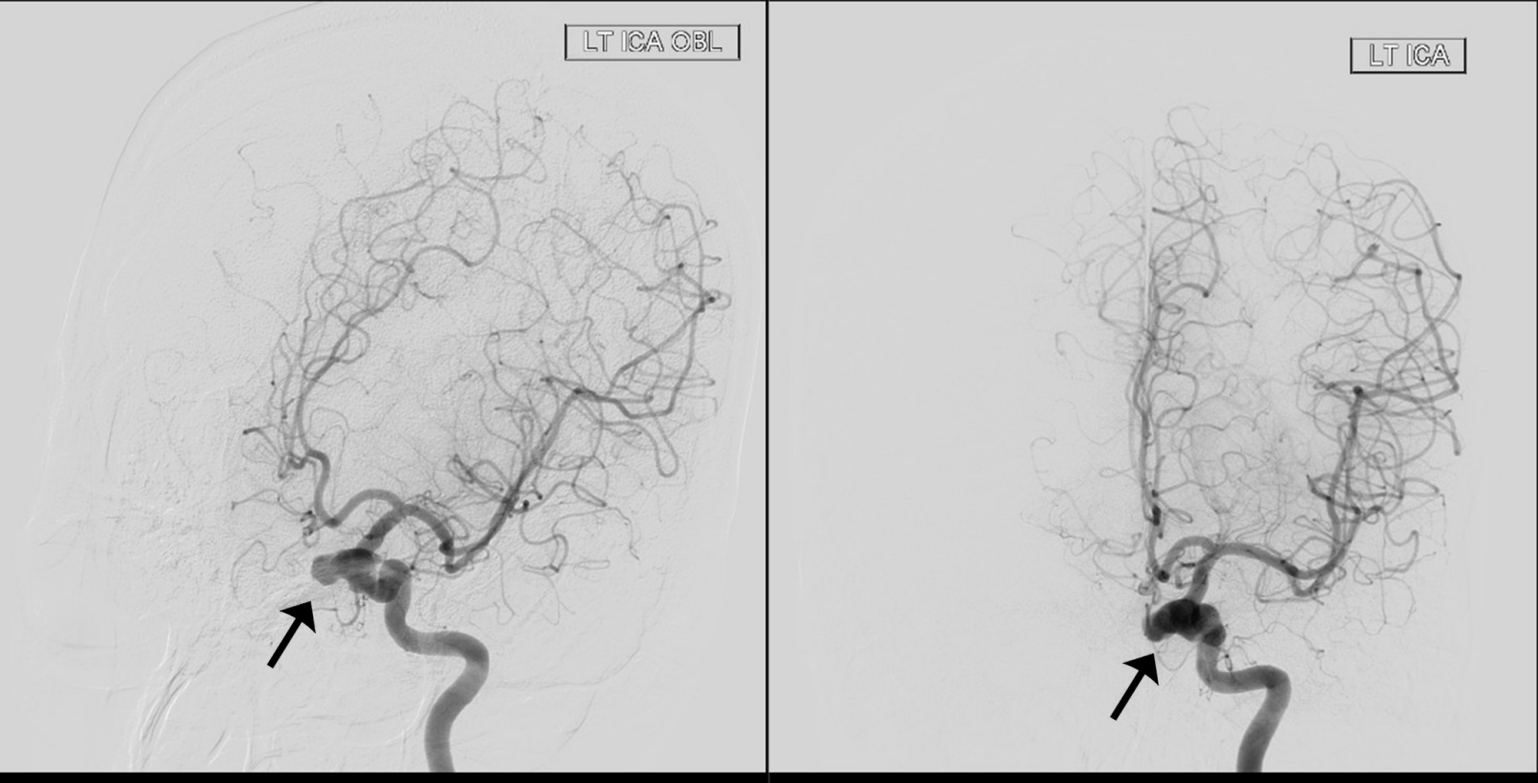
Preoperative digital subtraction angiogram (DSA) of the left internal carotid artery (ICA) (oblique and lateral views) showing aneurysm of the cavernous segment (black arrow).

On readmission with epistaxis he was severely pale and his hemoglobin levels had dropped to 5.6 g/dL. Medical and endonasal causes for epistaxis were initially ruled out. In view of his previous history of trauma, and the triad of blindness, epistaxis, and trauma a CT angiogram of the brain was performed, which revealed a saccular aneurysm of the left cavernous segment of the internal carotid artery (ICA). It was followed up with a digital subtraction angiogram (DSA) to confirm the flow and cross-circulation across the hemispheres and plan for surgical management. The DSA showed a large left cavernous segment pseudoaneurysm with moderate cross-circulation from the right ICA (Figure 1). There was no evidence of any fistulous connection.

**Figure 2:**
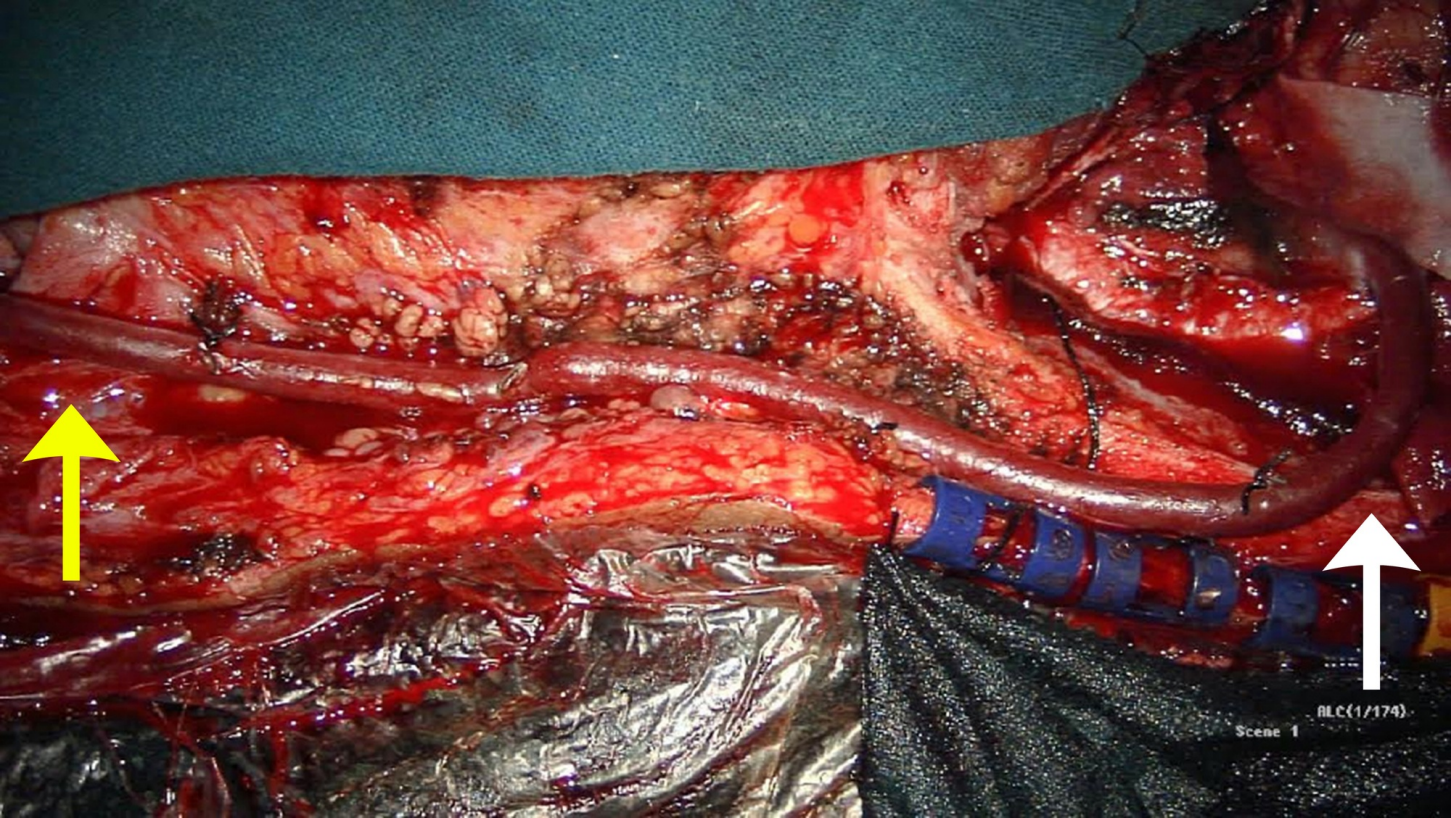
Interoperative image of the saphenous vein graft [extracranial-intracranial (EC-IC) bypass] – white arrow, cranial end; yellow arrow, carotid end.

The patient was explained about the need for intervention in the form of bypass surgery or endovascular flow diversion. They declined endovascular surgery due to financial constraints. In view of the suboptimal cross-circulation, it was decided to perform a replacement high-flow extracranial-intracranial (EC-IC) bypass and then trap the aneurysmal segment. A left pterional craniotomy and a high-flow EC-IC bypass (common carotid to M2 segment) with saphenous vein graft were performed followed by ligation of the left ICA in the neck (Figure 2).

**Figure 3:**
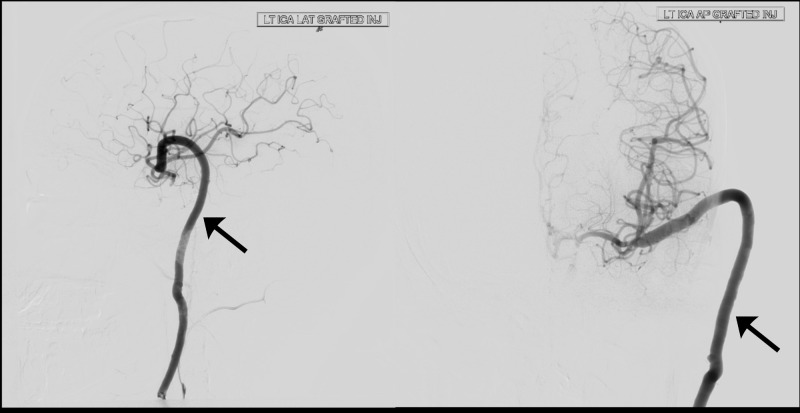
Postoperative digital subtraction angiogram (DSA) (AP and lateral views) showing good flow across the graft (black arrow) to the middle cerebral artery and anterior cerebral artery with no back flow into the aneurysm.

Postoperatively the patient was started on antiplatelets and had no complications. He made good postoperative recovery with daily monitoring of arterial pulsation and Doppler confirmation of the same. An angiogram taken on postoperative day nine revealed good flow across the bypass (Figure 3). The patient neither had any worsening of deficits, nor had any improvement in the vision of his left eye. He had no further episodes of epistaxis or any complaints at one year of follow up.

## Discussion

Cavernous carotid aneurysms (CCAs) are rare and account for only 2%–9% of all intracranial aneurysms and 15% of internal CCA [1]. The etiology of CCAs can be traumatic, infectious, or idiopathic. Parkinson classified aneurysms in this region as saccular and fistulous, and the fistulous variety can further be classified as spontaneous and traumatic [2]. Traumatic aneurysms are pseudoaneurysms consisting of a hematoma surrounded by a fibrous layer, rather than a true arterial wall, which rupture and cause massive epistaxis resulting from disruption through the sphenoid sinus wall [3-4]. Less commonly, pseudoaneurysms of the cervical ICA may rupture into the Eustachian tube or the posterior nasopharynx [5]. 

The natural history of idiopathic cavernous aneurysms is not well known. They often remain asymptomatic and they are detected incidentally. They tend to become large to giant in size when they manifest with features of mass effect in the form of cranial nerve palsies of adjacent nerves. This could be in the form of diplopia, ptosis, ophthalamoplegia, or pain or paresthesia along the fifth nerve distribution. Large transitional variant of these aneurysms, which has an intradural component can press against the optic nerve and result in visual symptoms. These intradural variants also carry a risk of subarachnoid hemorrhage (0.2%–0.4%) [6]. Rupture of a CCA more typically results in an arteriovenous (AV) fistula between the ICA and the cavernous sinus. Carotid cavernous fistulas can present with severe proptosis, chemosis, injection of the eye, pulsatile tinnitus, orbital bruit, and/or cranial nerve deficit. Rarely these aneurysms can erode into the sphenoid sinus and rupture resulting in fatal epistaxis. Spontaneous thrombosis of these aneurysms has also been reported as have been thromboembolic strokes originating from intra-aneurysmal thrombus [7].

The first cavernous segment aneurysm presenting with a triad of blindness, epistaxis, and trauma was described by Barth in 1924 [8]. Epistaxis is a relatively uncommon presentation of CCA, and is most often post-traumatic [9]. Nearly half of the patients present with epistaxis within the first month of head injury [10]. Presentation can, however, be delayed ranging from five days to nine weeks as in our patient who presented nearly four months after the head injury [3]. While cavernous segment ICA aneurysm is the most common cause of aneurysmal epistaxis, rupture of a superior hypophysis artery can also cause epistaxis [11]. Mortality from hemorrhage of such lesions has ranged as high as 50% [12]. Our patient lost valuable time and had a critically low hemoglobin value at presentation suggesting imminent complication. A high index of suspicion is required to identify and treat this condition. Clinically, the finding of the symptomatic triad of unilateral blindness, orbital fractures, and massive epistaxis is pathognomonic for ICA pseudoaneurysm [5].

Treatment modalities

Unlike idiopathic asymptomatic CCAs, traumatic, iatrogenic and infectious aneurysms have an aggressive course and need urgent intervention. Exclusion of the aneurysm from the circulation can be done either by surgery or endovascular techniques. Treatment can be either occlusive or reconstructive. Occlusive strategies include parent artery ligation surgically or by endovascular techniques. Reconstructive strategies include direct microsurgical clip application, coil embolization with or without the use of a vascular reconstruction device, flow-diverting devices, or the use of liquid embolic agents. Flow diverters have emerged as an excellent alternative to treat complex giant aneurysm where conventional methods have failed [13]. With the advent of flow diverters the results have improved with an obliteration rate of 60%–80% with morbidity of 10.44% (9.9%–15.2%) and mortality of 6.86% ( 2.3%–9.2%) [14]. However, in developing countries like India, the average cost of endovascular treatment is five to six times that of microsurgical bypass. This remains a major limitation for endovascular techniques and surgical option remains the main stay of treatment [15] as in the index case.

Surgery

Direct clipping probably provides the chance of near 100% obliteration maintaining parent vessel patency at the same time. However, literature provides reports of less than 150 cases of CCA, which have undergone direct clipping so far and even in experienced hands the morbidity and mortality rates are as high as 14%–25% [16]. Direct clipping is thus seldom preferred. 

Occlusive surgical options include proximal carotid ligation with or without trapping the aneurysmal carotid segment by placing a clip proximal to the ophthalmic artery. Proximal carotid ligation carries a risk of ischemia to the ipsilateral hemisphere. It also carries a risk of new aneurysm formation on the contralateral side or increase in the size of any contralateral aneurysm if any. Risk of ischemia can be predicted by various techniques like rate of venous filling, balloon test occlusion (BTO), single-photon emission computed tomography (SPECT), and positron emission tomography (PET). The risk of infarct is 32%–60% if carotid ligation is carried out without any preoperative assessment of cerebrovascular reserve [17-18]. The risk of infarction comes down to 22% in patients who successfully complete all preoperative assessment tests [19]. The risk, however, does not completely disappear. With an additional bypass this risk comes down to 14.6% (1.8%–29.4%) [19]. A bypass procedure prior to parent artery occlusion is, therefore, preferable to reduce the risks of postocclusion stroke even in patients who tolerate BTO successfully (universal bypass). 

Bypass could be an augmentative (low flow superficial temporal-middle cerebral) bypass for patients with moderate cerebrovascular reserve or a replacement bypass (high-flow extracranial–intracranial) for those with poor cerebrovascular reserve. Similarly the choice between parent artery occlusion alone or trapping is difficult as it has been shown that there is no difference in complications and outcome between trapping and carotid occlusion [18]. Trapping is preferred to mere carotid occlusion for aneurysms with significant intradural extension and patients who demonstrate significant retrograde flow during the BTO [20].

Our patient presented with unilateral blindness and epistaxis justifying treatment. We did not perform a BTO, SPECT, or PET study for our patient as the cross-compression study revealed less than adequate cross-circulation. Saphenous vein graft and common carotid artery for proximal anastomosis were chosen due to surgeon’s preference and past experience. Our patient successfully underwent a high-flow bypass procedure and ligation of the internal carotid. 

## Conclusions

Traumatic CCAs are rare and pose considerable challenges in diagnosis and management. A high index of suspicion should be kept in all patients with skull base fractures near the optic canal, cavernous sinus. Endovascular treatment strategies offer reasonable results, but are expensive. Parent artery occlusion combined with a universal bypass is a cost-effective alternate option with comparable results.
